# Sodium Hydrogen Exchanger Regulatory Factor-1 (NHERF1) Regulates Fetal Membrane Inflammation

**DOI:** 10.3390/ijms21207747

**Published:** 2020-10-20

**Authors:** Ananth Kumar Kammala, Samantha Sheller-Miller, Enkhtuya Radnaa, Talar Kechichian, Hariharan Subramanian, Ramkumar Menon

**Affiliations:** 1 Department of Obstetrics and Gynecology, Division of Maternal-Fetal Medicine and Perinatal Research, The University of Texas Medical Branch at Galveston, 301 University Blvd., Galveston, TX 77555-1062, USA; ankammal@utmb.edu (A.K.K.); sashelle@utmb.edu (S.S.-M.); enradnaa@utmb.edu (E.R.); takechic@utmb.edu (T.K.); 2 Department of Physiology, Michigan State University, 567 Wilson Rd, East Lansing, MI 48824, USA; subram46@msu.edu

**Keywords:** NHERF1, amniochorion, infection, preterm birth, NF-kB, interleukin-10, interleukin-6, interleukin-8 pregnancy, parturition

## Abstract

The fetal inflammatory response, a key contributor of infection-associated preterm birth (PTB), is mediated by nuclear factor kappa B (NF-kB) activation. Na^+^/H^+^ exchanger regulatory factor-1 (NHERF1) is an adapter protein that can regulate intracellular signal transduction and thus influence NF-kB activation. Accordingly, NHERF1 has been reported to enhance proinflammatory cytokine release and amplify inflammation in a NF-kB-dependent fashion in different cell types. The objective of this study was to examine the role of NHERF1 in regulating fetal membrane inflammation during PTB. We evaluated the levels of NHERF1 in human fetal membranes from term labor (TL), term not in labor (TNIL), and PTB and in a CD1 mouse model of PTB induced by lipopolysaccharide (LPS). Additionally, primary cultures of fetal membrane cells were treated with LPS, and NHERF1 expression and cytokine production were evaluated. Gene silencing methods using small interfering RNA targeting NHERF1 were used to determine the functional relevance of NHERF1 in primary cultures. NHERF1 expression was significantly (*p* < 0.001) higher in TL and PTB membranes compared to TNIL membranes, and this coincided with enhanced (*p* < 0.01) interleukin (IL)-6 and IL-8 expression levels. LPS-treated animals delivering PTB had increased levels of NHERF1, IL-6, and IL-8 compared to phosphate-buffered saline (PBS; control) animals. Silencing of NHERF1 expression resulted in a significant reduction in NF-kB activation and IL-6 and IL-8 production as well as increased IL-10 production. In conclusion, downregulation of NHERF1 increased anti-inflammatory IL-10, and reducing NHERF1 expression could be a potential therapeutic strategy to reduce the risk of infection/inflammation associated with PTB.

## 1. Introduction

Pregnancy is a state of immune homeostasis where balanced inflammation at various tissue levels promote fetoplacental growth [1,2,3]. Increased inflammation and/or an imbalance in immune homeostasis in various intrauterine tissues are among the key mediators of parturition in humans as well as in animals. The well-characterized inflammatory mediators are pro- and anti-inflammatory cytokines, chemokines, and growth factor concentration changes in various fetomaternal tissues [4]. A proinflammatory shift is required to transition quiescent uterine tissues to an active prolabor state [5]. A multitude of signals from both the mother and fetus coordinate to generate inflammatory mediators. These mediators increase decidual inflammation, cause cervical ripening, and induce contractility of the myometrium on the maternal side as well as lead to a dysfunctional fetal membrane that functions to maintain uterine architecture during pregnancy [6]. Understanding the mechanisms that regulate acute changes in inflammatory mediators is critical to reduce the risk of spontaneous preterm birth (PTB) and preterm premature rupture of membranes (pPROM), two major complications of pregnancy and conditions associated with inflammation [3].

Inflammation often involves activation of the master transcription factor nuclear factor kappa B (NF-kB). Infection and other proinflammatory stimuli-induced NF-kB activation is mediated by several factors [7,8,9,10,11]. One of the factors that can influence NF-kB activation in a cell is Na^+^/H^+^ exchange regulatory factor-1 (NHERF1). NHERF1 is an adapter protein that binds to several G-protein coupled receptors (GPCR), regulates signal transduction complexes, and mediates cytoskeletal–plasma membrane interactions [12]. NHERF proteins can also regulate cellular responses in a GPCR-independent manner by downstream signaling proteins [13]. Multiple studies have shown various mechanisms of NHERF1’s involvement in NF-kB activation. For example, NHERF1 is a positive regulator of NF-kB activation in vascular inflammation by recruiting PKCζ at the cell membrane, resulting in activation of NF-kB [14]. NHERF1 maintains cyclic amino monophosphate (cAMP) and PKA signaling and regulates interleukin (IL)-6 production in human airway smooth muscle cells [15] and promotes mast cell activation via the C3a complement receptor (C3aR) pathway [16].

Inflammation during parturition is mediated via several mechanisms. Multiple studies have shown involvement of the mitogen-activated protein kinase (MAPK) pathway [17]. Using fetal membranes as a model to test fetal inflammatory response, our laboratory has shown that term and preterm parturition is associated with p38 MAPK activation, resulting in localized inflammation through either cellular senescence or cellular transitions [18,19,20]. Others have shown that the extracellular receptor kinase (ERK) and Janus kinase (JNK) pathways in membranes produce inflammation at term and preterm parturition [21]. Regardless of signaling pathways, NF-kB activation and an increase in proinflammatory mediators are the final effectors of parturition [22,23,24,25]. This is a natural and physiological response at term, but is pathologic and prematurely activated in preterm parturitions.

As mentioned above, NHERF1 is an intermediary between various signaling molecules and affects proinflammatory cytokine production; however, the expression profile of this molecule is not well characterized in fetal membrane tissues or mechanistically shown to have a functional impact on fetal membrane inflammation. Therefore, we tested the hypothesis that NHERF1 promotes NF-kB activation in response to an infectious stimulus and that regulation of NHERF1’s function can reduce fetal membrane inflammation associated with term and PTB. Using fetal membranes from term and preterm birth and primary cells from fetal membranes, this study determined NHERF1’s differential expression and signaling and its role in generating inflammatory mediators in response to an infectious stimulus.

## 2. Results

### 2.1. NHERF1 Levels Are Upregulated in Fetal Membranes of TL and PTB

**Figure 1:**
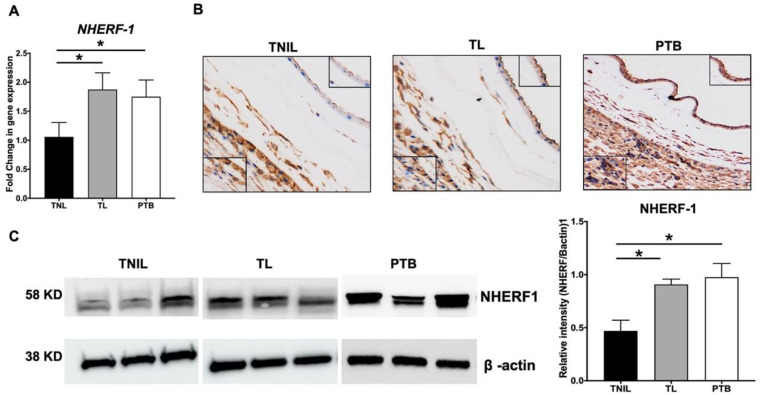
Expression and localization of Na^+^/H^+^ exchanger regulatory factor-1 (NHERF1) in human fetal membranes. (**A**) Fetal membrane tissues from term not in labor (TNIL), term labor (TL), and preterm birth (PTB) were analyzed for gene expression levels of NHERF1. Data is shown as mean fold change ± SEM with a total of 10–12 samples per cohort. (**B**) Immunohistochemistry (IHC) images of TNIL, TL, and PTB fetal membranes stained for the NHERF1 protein is shown. Representative images from a minimum *n* = 3 for each tissue type are shown. NHERF1 was found localized to both chorion and amnion layers of fetal membranes. (**C**) Western blotting (WB) was performed to determine NHERF1 levels in TNIL, TL, and PTB fetal membranes. The blots were stripped and reprobed with β-actin as a loading control. The relative levels of NHERF1 increased two-fold in PTB and TL compared to TNIL samples. Representative blots from *n* = 3 for each tissue type are shown. Data are mean ± SEM of three independent experiments. Statistical significance was determined by one-way ANOVA. * *p* < 0.05 vs. TNIL.

Fetal membranes are broadly divided into morphologically distinct layers, the amnion membrane and chorion membrane, each with its own well-defined microarchitecture [26]. To determine differential expression of the NHERF1 gene and its localization in different layers of fetal membranes, qRT-PCR and IHC were performed. Interestingly, NHERF1 transcripts were significantly elevated (*p* < 0.01) in fetal membranes from TL and PTB compared to TNIL (*p* < 0.01), as shown in Figure 1A. NHERF1 localized in Amnion and chorion layers of fetal membranes, regardless of pregnancy status (Figure 1B); i.e., TL, TNIL and PTB. Although not quantitatively assessed, the intensity of NHERF1 staining was distinctly higher in the amnion layer of membranes from TL and PTB (Figure 1B; see inset top right). Consistent with the quantitative real-time PCR (qRT-PCR) and immunohistochemistry (IHC) data, NHERF1 protein levels as determined by western blotting (WB) were significantly (*p* < 0.01) higher in TL and PTB, as shown in Figure 1C. As fetal membrane samples used for this study were deidentified, we were unable to perform any stratified analysis based on the specific types of risk factors associated with PTB. It is likely that infectious and noninfectious factors that cause inflammation may have contributed to PTB in our cohort, and fetal membrane sterile inflammation is also a trigger for TL.

### 2.2. NHERF1 Increase Is Associated with Proinflammatory Cytokine Switch in Fetal Membranes

**Figure 2:**
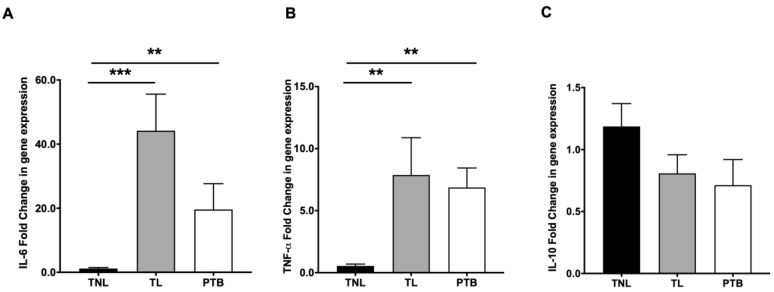
Levels of proinflammatory cytokines in human fetal membranes. Fetal membrane tissues from TNIL, TL, and PTB were analyzed for gene expression levels of (**A**,**B**) proinflammatory cytokines, such as interleukin (IL)-6 and IL-8, and (**C**) anti-inflammatory cytokine IL-10 levels. Data is shown as mean fold change ± SEM with a total of 8–12 samples per cohort. Statistical significance was determined by one-way ANOVA. *** *p* ≤ 0.001 and ** *p* ≤ 0.01 vs. TNIL.

To further confirm inflammation in TL and PTB membranes, we tested differential expressions of proinflammatory cytokines that are NF-kB responders, such as IL-6 and tissue necrosis factor-α (TNF-α), as well anti-inflammatory cytokines, such as IL-10 [27], in our specimens. We found a significant (*p* < 0.01) increase in the gene expression levels of IL-6 (Figure 2A) and IL-8 (Figure 2B) and a modest decrease in the expression of anti-inflammatory cytokine IL-10 in TL and PTB fetal membranes (Figure 2C).

### 2.3. NHERF1 Levels Were Enhanced in the Fetal Membranes of Mice Subjected to PTB

**Figure 3:**
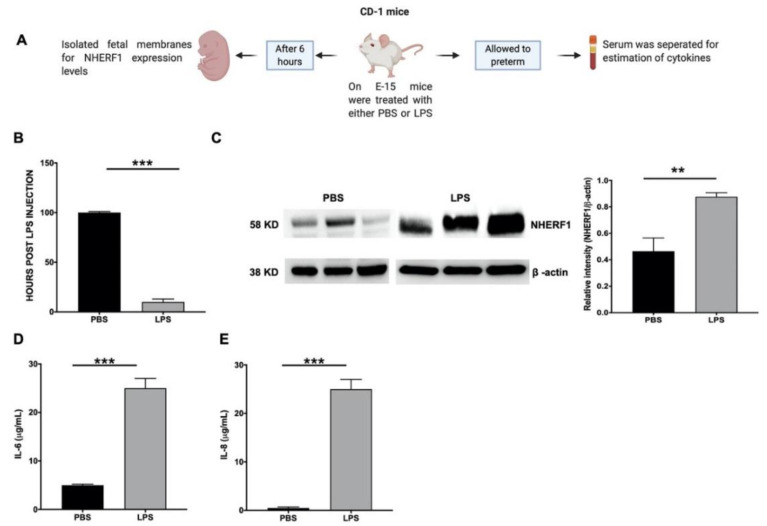
Expression of NHERF1 in lipopolysaccharide (LPS)-induced murine model for preterm birth. (**A**) Schematic representation of LPS-induced preterm birth model is shown. (**B**) Survival rate graph of LPS-induced pregnant mice. (**C**) Fetal membranes from cohorts of mice that were exposed to phosphate-buffered saline (PBS) or LPS were subjected to western blot analysis to determine NHERF1 levels. (**D**,**E**) The maternal serum was collected for the estimation of proinflammatory cytokines IL-6 and IL-8. Data is shown as mean fold change ± SEM with a total of six samples per cohort. Statistical significance was calculated using an unpaired student’s *t*-test *** *p* ≤ 0.001 and ** *p* ≤ 0.01.

In the next step, we determined whether NHERF1 expression was upregulated in inflammation-associated preterm labor in vivo. We adopted a previously described model of LPS-induced PTB that is dependent on NF-kB activation [28]. CD1 mice were injected with either lipopolysaccharide (LPS) or phosphate-buffered saline (PBS) on E15. A schematic diagram of the experimental model is shown in Figure 3A. LPS-treated mice delivered preterm within 24 h compared to PBS-injected animals (*p* < 0.01) (Figure 3B). In a subset of animals, NHERF1 expression and inflammatory cytokines were assessed in fetal membranes 6 h after LPS treatment. Fetal membranes from these mice were also subjected to western blotting to detect protein levels of NHERF1. The representative blots are shown in Figure 3C. NHERF1 levels were significantly (*p* < 0.01) upregulated in fetal membranes from mice treated with LPS compared to mice injected with PBS. Additionally, maternal serum inflammatory markers IL-6 (Figure 3D) and IL-8 (Figure 3E) were significantly increased in LPS-treated mice.

In summary, increased expression of NHERF1 was associated with TL and PTB, coinciding with an increase in inflammatory cytokines. Although our data suggest an overall increase in NHERF1 in fetal membranes in both human and mouse samples, membrane cell type-specific NHERF1 expression was not determined from these experiments. Therefore, the remaining studies were conducted using primary cells derived from fetal membranes.

### 2.4. NHERF1 Levels Are Enhanced in Different Cell Layers of Fetal Membranes Following LPS Stimulation

**Figure 4:**
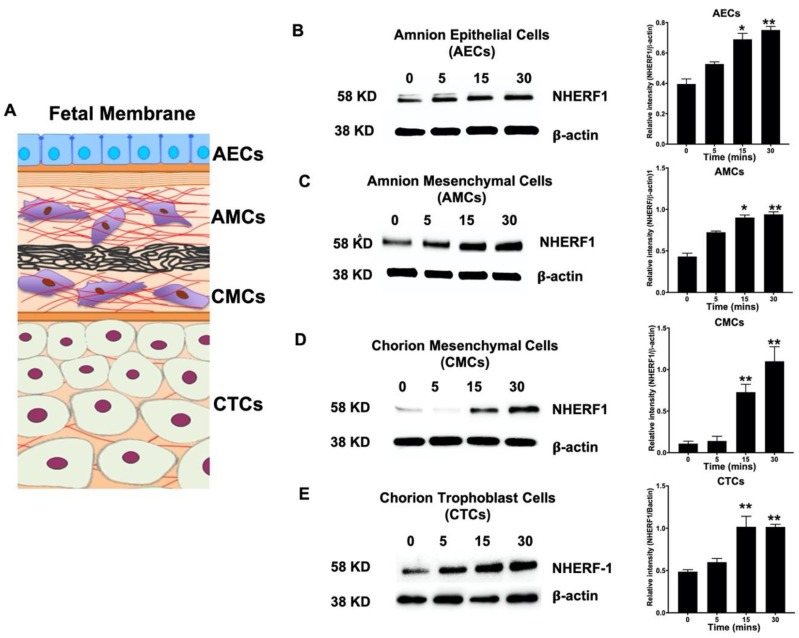
Expression levels of NHERF1 in human fetal membrane primary cells induced by LPS. (**A**) Representation of different layers of human fetal membrane. WB analysis of (**B**) amnion epithelial cells (AECs), (**C**) amnion mesenchymal cells (AMCs), (**D**) chorion mesenchymal cells (CMCs), and (**E**) chorion trophoblast cells (CTCs) to determine the levels of NHERF1 in response to LPS at different timepoints. The relative levels of NHERF1 significantly increased in a time-dependent manner with LPS treatment compared to the control in different layer cells of fetal membrane. The blots were stripped and reprobed with β-actin as a loading control. Bar graphs show relative intensities of bands for the NHERF1 protein for respective primary cells. Representative blots from *n* = 3 for each cell type are shown. Data presented as mean ± SEM. Statistical significance was determined by one way ANOVA. * *p* < 0.05, ** *p* ≤ 0.01 vs. 0 min.

LPS induces downstream signaling that ultimately translocates active NF- kB into the nucleus, resulting in cytokine production [29]. To determine the role of NHERF1 in primary cells of fetal membrane during inflammation, we exposed primary cells of fetal membranes to LPS for different time intervals (0, 5, 15, and 30 min) and analyzed NHERF1 expression by western blotting. NHERF1 levels were significantly upregulated in all different types of primary cells—amnion epithelial cells (AECs), amnion mesenchymal cells (AMCs), chorion mesenchymal cells (CMCs), and chorion trophoblast cells (CTCs)—following LPS stimulation (*p* > 0.05) at different time intervals, as shown in Figure 4B–E.

### 2.5. Translocation of NHERF1 in Amnion Epithelial Cells with LPS Stimulation

**Figure 5:**
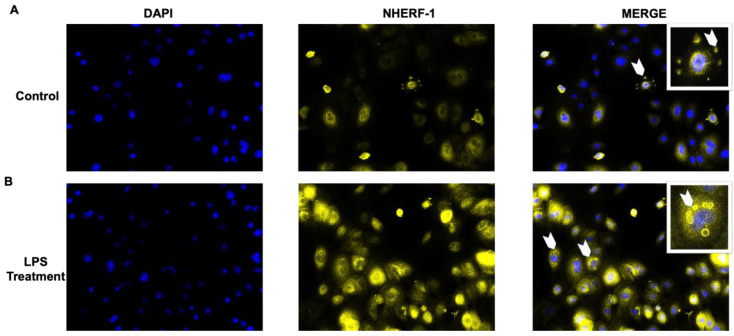
Translocation of NHERF1 upon LPS stimulation in AECs. AECs were exposed to (**A**) vehicle or (**B**) LPS (100 ng/mL) for 15 min, washed, and stained for NHERF1 expression (**yellow**) using immunofluorescence (IF). 4,6-diamidino-2-phenylindole (DAPI; **blue**) staining was performed to detect the nucleus. Representative images are shown. Scale bar = 10 µm. Fluorescent images were collected with an Olympus BX43 fluorescence microscope at 40× magnification. The inset image is of an enlarged area marked by white arrows showing special organelle translocation towards the nucleus to LPS stimulation.

To directly visualize and determine NHERF1 localization in primary cells, immunofluorescence (IF) staining for NHERF1 was performed after 15 min of LPS treatment. We observed intense NHERF1 staining in the membrane of intracytoplasmic organelles, which are likely lumens and microlumens in cells exposed to LPS [30,31]. As shown in Figure 5, NHERF1 formed round clusters (yellow rings; see small arrows) on lumens and translocated close to the nucleus after treatment. This localization was substantially higher after 15 min of LPS treatment (bottom panel; see inset in merged image). The functional role of NHERF1 clusters around lumen in fetal membrane cells is currently unclear; however, as reported previously in other cell types [32], it may represent an activation state of cellular biogenesis.

### 2.6. Silencing NHERF1 in Primary Cells Results in a Reduction in Phospho-NF-kB Levels and Attenuates IL-6 and IL-8 Production

**Figure 6:**
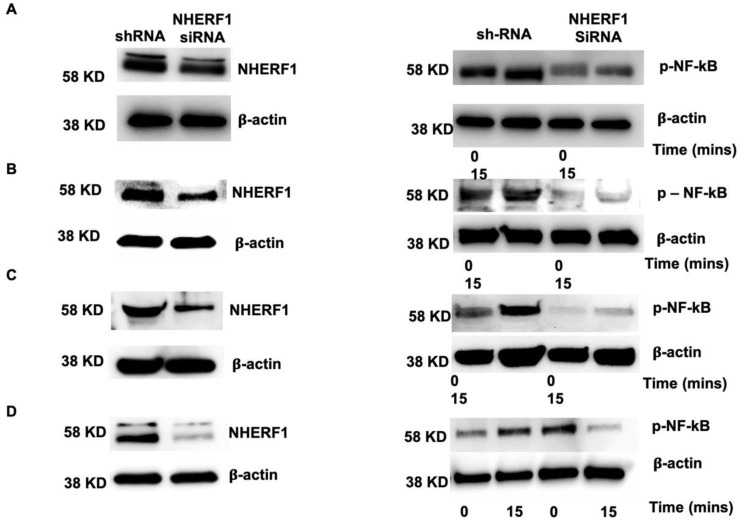
Silencing NHERF1 expression results in decreased p-NF-kB levels. Primary fetal membrane cells (**A**) AECs, (**B**) AMCs, (**C**) CMCs, (**D**) and CTCs were transiently transduced with NHERF1 small interfering RNA (siRNA) and nontargeted siRNA as a control. Representative blots of NHERF1 knockdowns is shown (left column) for respective primary cell type. NF-kB levels following activation with LPS for 15 min is shown in the right column. Representative blots for four different types of primary cells from three independent experiments are shown. The blots with NHERF1 and p-NF-kB were stripped and reprobed with the β-actin antibody for loading control.

**Figure 7:**
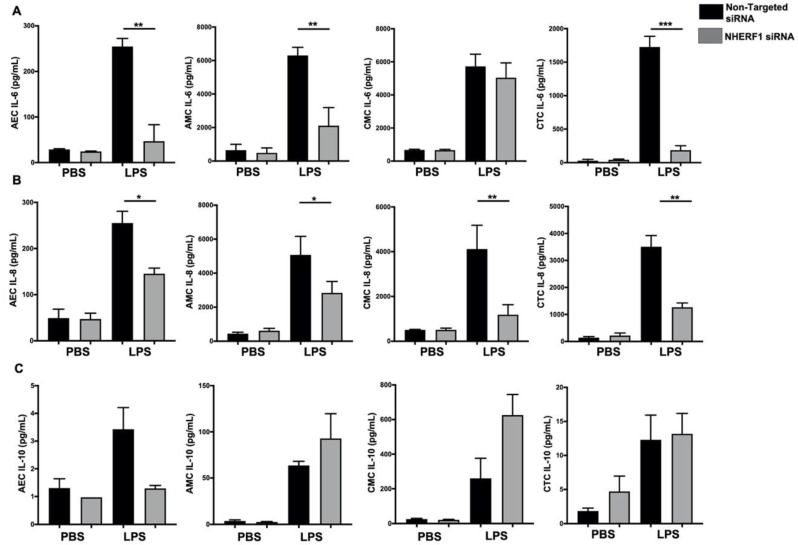
Effect of NHERF1 in production of inflammatory cytokines from primary cells treated by LPS. Control nontargeted siRNA and NHERF1 siRNA knockdown cells were treated with LPS for 48 h, and a multiplex assay was performed to estimate (**A**) IL-6, (**B**) IL-8, and (**C**) IL-10 levels in different primary cell supernatants. Data is mean ± SEM from three experiments. Statistical significance was determined by unpaired student *t*-test considering scrambled RNA as a control. * *p* < 0.05, ** *p* < 0.01, and *** *p* < 0.001.

To confirm that NHERF1 functions to regulate NF-kB activation in cells exposed to LPS, we conducted additional experiments where NHERF1 expression was silenced using transient transfection of NHERF1-specific small interfering RNA (siRNA). We used nontarget siRNA-transduced cells as controls. NHERF1 expression levels were reduced (by ~80%) as determined by western blot analysis (Figure 6A–D left column). As expected, p-NF-kB levels were substantially reduced after 15 min of LPS stimulation in NHERF1 knockdown cells compared with nontarget siRNA-treated cells (Figure 6A–D, right column). Furthermore, downregulation of NHERF1 expression significantly decreased proinflammatory cytokine (IL-6 and IL-8) production and trend towards increased anti-inflammatory IL-10 production, as shown in Figure 7A–C. These data suggest that NHERF1 regulates p-NF-kB and proinflammatory cytokine levels following LPS stimulation of fetal membrane cells.

## 3. Discussion

Human parturition, at term or preterm, is an inflammatory process. Infectious or noninfectious inflammation contributes to over 70% of all PTB and neonatal morbidities [3]. NF-kB is a pleiotropic transcription factor that increases proinflammatory cytokine production, and regulation of its activation is therefore considered one of the approaches to reduce the risk of PTB [33,34,35]. Novel therapeutics that reduce inflammation by inhibiting NF-kB using anti-inflammatory drugs [36,37,38], cell-penetrating peptides, small molecule inhibitors [33,39,40], nonspecific inhibitors [41,42,43,44], and flavonoids are now being tested [45,46]. However, very few have made it to clinical trials so far and none are in use clinically, likely due to several issues including, but not limited to, the mode of delivery, placental permeability, teratogenicity, and transgenerational effects. One approach to improve pregnancy outcomes is to find upstream regulators or downstream mediators of NF-kB as alternate targets for intervention. In this study, we examined the role of NHERF1, one of the upstream regulators of NF-kB, in regulating NF-kB transcription in primary cells from human fetal membranes. Our principle findings suggest that increased expression of NHERF1 is associated with NF-kB activation, and its regulation can control proinflammatory cytokine release from fetal membrane cells. More importantly, downregulation of NHERF1 increases anti-inflammatory IL-10, a cytokine that has been shown to inhibit preterm contractions in various animal models in response to infection and inflammation [47,48,49].

The upregulation of NHERF1 has been reported in multiple disease states, including hepatocellular carcinomas, cholangiopathies, glioblastoma, breast cancer, psoriasis, and vascular injury [50,51,52,53,54,55,56]. Although it has been well established that NHERF1 upregulates NF-kB signaling, its role in fetal membrane-induced inflammation is not clear [25,57,58]. This is the first report to determine the role of NHERF1 in fetal membrane inflammation and its potential contribution to infection-associated PTB and pPROM. Typically, NHERF1 is a PDZ-scaffolding protein that organizes functional complexes and modulates kinase activities to accurately coordinate specific signaling pathways [59]. Previous studies have shown that NHERF1 expression is increased in inflammatory carcinogenic tissues [60,61]. Similarly, we observed higher NHERF1 gene expression in human fetal membranes from TL and PTB that are triggered by inflammation compared to membranes not in labor. Consistent with this data, we also observed increased NHERF1 protein expression in TL and PTB membranes. NHERF1 protein expression was positively correlated with proinflammatory cytokines and negatively correlated with IL-10. Thus, our study suggests that infection-induced upregulation of NHERF1 increases NF-kB and subsequently increases overall inflammation. Additionally, we observed a unique pattern of expression of NHERF1 in intraluminal vesicles in cells after LPS treatment, which is indicative of cellular activation. As NHERF1 is involved in NF-kB activation, it is likely that NHERF1 interacts with late endosomes and is involved in the endosomal protein trafficking pathway to reach mitochondria and execute multitudes of functions [32]. This suggests that reducing NHERF1 is an ideal target for regulating infection/inflammation-associated preterm labor as it can downregulate NF-kB and increase IL-10.

Our data agrees with several other reports of LPS-induced inflammation and PTB in a mouse model [61,62,63]. In addition, we report that this inflammation is also associated with elevated NHERF1. An interesting finding in our study is related to the increase in IL-10 when NHERF1 was downregulated. Using an IL-10 knockout model, Sarah et al. reported that IL-10 mediated downregulation of proinflammatory cytokines in the uterus and placenta, which was required to prevent PTB in an LPS-induced mouse model [64]. Similarly, Sadowski et al. showed that IL-10 delayed IL-1b-induced PTB in nonhuman primate models [65]. Multiple in vitro reports by Fortunato and Menon showed that IL-10 inhibited proinflammatory cytokine production and MMP activation in in vitro human fetal membrane explants [66,67]. Thus, the possibility of NHERF1 as an interventional target is intriguing as its downregulation can increase the presence of anticontractile IL-10.

The NF-kB plays an essential role in the regulation of immune and inflammatory responses [67]. Our experiments focus on the role of NHERF1 in the regulation of p-NF-kB levels and cytokine production in fetal membrane primary cells. Our lab has demonstrated that other signaling molecules, such as p38, MAPK, and glycogen synthase kinase 3-β (GSK3β), are involved in the establishment and maintenance of inflammatory conditions by the fetal membranes [16]. Recent studies have highlighted the emerging role of NHERF1 in mediating inflammation. Leslie et al. showed that NHERF1 expression increased upon TNF-α treatment in primary macrophages and vascular smooth muscle cells. Conversely, deletion of NHERF1 resulted in impaired activation of NF-kB in these cells. In NHERF1^−/−^ mice, macrophage activation, and recruitment to vascular lesions as well as vascular inflammation were reduced after LPS treatment. In the same study, the authors also reported that inflammatory stimuli promote the formation of an NHERF1–PKCζ complex in the cell membrane that induces NF-kB signaling. Thus, NHERF1 and NF-kB participate in a feed-forward loop, leading to increased macrophage activation and enhanced response of vascular cells to inflammation [14]. It remains to be determined if this feed-forward loop also regulates the inflammatory response in fetal membranes. Regardless, NHERF1 and NF-kB axis is critical in generating fetal membrane inflammation in response to an infectious stimulus.

Another role for NHERF1 is associated with determining cell fate in response to a stimulant. For example, Wnt signaling is associated with G-protein-coupled receptors, named Frizzled receptors, that activate the downstream pathway by MAPK and GSK3β [68,69]. The GSK3β pathway maintains the cell cycle in human fetal membranes. We have reported that downregulation of GSK3β by its phosphorylation, coupled with activation of stress signaler p38 MAPK, is a mechanism associated with fetal membrane stress, senescence, and inflammation [68,70]. We postulate that LPS-mediated activation of p38 MAPK [71] and other kinases [72] may downregulate GSK3β and decrease pro cell growth signaling. In such cells, NHERF1 activation could be a by-product of cell cycle arrest.

NHERF1 levels were augmented during LPS-mediated inflammation, and inflammation was reduced when NHERF1 was silenced. These results are consistent with previous reports that have demonstrated NHERF1 knockdowns abrogate LPS-induced cAMP-mediated IL-6 production in human airway smooth cells [15]. Similarly, inhibition of NHERF1-CXCR2 has been shown to prevent chemotaxis and neutrophil adhesion driven by IL-8 [73]. In summary, we have demonstrated NHERF1’s mediation in increasing fetal membrane inflammation via NF-kB during in-term and preterm parturition. Further, NHERF1 knockout models can demonstrate its functional contributions in infection associated PTB and its usefulness as an interventional target to reduce inflammation. Downregulation of NHERF1 increased anti-inflammatory mediator IL-10, indicating that reducing NHERF1 expression could be a potential therapeutic strategy to reduce the risk of infection/inflammation associated with PTB.

## 4. Materials and Methods

### 4.1. Institutional Review Board (IRB) Approval

Placentae for this study were collected after term vaginal or cesarean deliveries (TL and TNIL) and preterm if deliveries were below 37 weeks from John Sealy hospital (University of Texas Medical Branch (UTMB)) at Galveston, Texas, according to the inclusion and exclusion criteria defined by our laboratory. As discarded placentas were used after delivery for the study, subject recruitment and/or consent was not collected. The Institutional Review Board at UTMB approved the study protocol with IRB approval number IRB16.0058, January 2020. and placentas were collected according to the regulations of the IRB as an exempt protocol that allowed utilization of the discarded unidentified placentae.

### 4.2. Clinical Samples

Fetal membranes (amniochorion) were obtained from TNIL (*n* = 12), TL (*n* = 10), and PTB (*n* = 10), as described previously by our laboratory [74]. Briefly, fetal membranes were dissected from the placenta, and any adherent blood clots were removed by washing the membranes in normal saline, followed by thorough cleaning using sterile cotton gauze. Biopsies of 6 mm (explants) were collected from the midzone of the fetal membranes. Intact fetal membrane explants (amniochorion) as well as explants with the amnion and chorion layer separated were collected and processed to perform IHC and WB.

### 4.3. Quantitative Real-Time PCR for NHERF1 and Cytokines

Fetal membranes were dissociated in Trizol (Life Technologies, Carlsbad, CA, USA) using a high-speed homogenizer. RNA was isolated using an RNeasy Kit (QIAGEN, Hilden, Germany) and treated with RNase-free DNase (QIAGEN). cDNA was synthesized using a High-Capacity cDNA Reverse Transcription Kit (Thermo Fisher, Waltman, MA, USA) according to the manufacturer’s instructions. Real-time qRT-PCR was performed using validated TaqMan gene expression NHERF1 primer (assay ID: Hs00188594_m1, gene symbol: SLC9A3R1), IL-10 primer (assay ID: Hs00961622_m1, gene symbol: IL-10), IL-6 primer (assay ID: Hs00174131_m1, gene symbol: IL-6), and TNF-α (assay ID: Hs00174128_m1, gene symbol: TNF) and probe sets according to the manufacturer’s instructions. qRT-PCR reactions were carried out using Bio-Rad CFX PCR Instruments (Bio-Rad, Hercules, CA, USA). Results were analyzed using CFX maestro software. mRNA expression was calculated using the 2^−ΔCt^ method with GAPDH as an internal reference control.

### 4.4. Immunohistochemistry for NHERF1

IHC for NHERF1 was performed on fetal membrane samples from TL, TNIL, and PTB using methods previously described by our laboratory [74]. Briefly, fetal membrane sections were fixed for 48 h in 4% paraformaldehyde (PFA) and embedded in paraffin. Tissue sections of 5 μm thickness were cut and adhered to a positively charged slide. Xylene was used to deparaffinize the tissues, and 100% alcohol, 95% alcohol, and normal saline (pH 7.4) were used for rehydration before proceeding to staining. Tissue sections were probed with the antihuman NHERF1 antibody (1:200, ab3452, Abcam, Cambridge, UK) overnight at 4 °C, and IHC antirabbit (1:500, Vector Laboratories, CA, USA) was added for 1 h at room temperature, followed by DAB as a chromogen for 5 min and hematoxylin as a counter stain for color development. Five images for each specimen were taken at 20× and 40× magnification.

### 4.5. In Vivo Murine Model for Preterm Birth

Animal care: All animal procedures were approved by the Institutional Animal Care and Use Committee (IACUC) at the University of Texas Medical Branch, Galveston. The approved animal protocol number was 0411077E dated on October 2019. Timed-pregnant CD1 mice (Charles River Laboratories, Houston, TX, USA) were received on gestational day (E) 14. Mice were housed in a temperature- and humidity-controlled facility with automatically controlled 12:12 h light and dark cycles with regular chow and drinking solution provided ad libitum. Animal care and regular maintenance was provided by certified personnel and veterinary staff according to IACUC guidelines. Animals were sacrificed by CO_2_ inhalation before tissue collection according to IACUC and American Veterinary Medical Association guidelines.

### 4.6. LPS-Induced PTB

On E15, pregnant dams were intraperitoneally (IP) injected with either PBS or lipopolysaccharide (serotype 055: B5, Sigma-Aldrich, St. Louis, MO (100 µg in 100 uL)) [75]. Animals were monitored for preterm delivery, which was defined as delivery of at least one pup on or before E18.5 (normal term is E19–E21 in this strain) using Wansview Wireless cameras (Shenzhen, China). A subset of CD1 mice were euthanized at the time of LPS-induced preterm labor (14 h post injection), and plasma was collected for cytokine analysis.

### 4.7. Luminex Assay to Determine Cytokine Concentration in Maternal Plasma

Plasma collected from mice were assayed for IL-10, IL-6, and IL-8 (*n* = 3 per group) using MILLIPLEX Mouse Cytokine Panel 1 (Millipore) following the manufacturer’s protocol. Standard curves were developed using duplicate samples of known-quantity recombinant proteins that were provided by the manufacturer. Sample concentrations were determined by relating the absorbance of the samples to the standard curve using linear regression analysis.

### 4.8. Isolation and Culture of Human Amnion Epithelial Cells and Human Amnion Mesenchymal Cells

To determine the cell-type-specific effect of NHERF1 response, primary cells from fetal membranes were isolated.Human AEC were isolated and cultured as previously described by our laboratory [74,75,76]. For preparing primary cells, we collected a total of 12 placentae for this study. Concisely, about 10 g of the amnion layer from the TNIL placenta was parted from the chorion layer.The amnion was cleaned in saline and cut into small pieces approximately 2 cm × 2 cm in size, followed by digestion using 0.125% collagenase and 1.2% trypsin (Sigma-Aldrich, St. Louis, MO, USA) in Hanks’ balanced salt solution (HBSS; Mediatech Inc., Manassas, VA, USA) for 35 min. After the digestion step, the tissue was filtered through a 70 µm cell strainer (Thermo Fisher Scientific, Waltham, MA, USA), and trypsin was inactivated with AEC culture media. The complete AEC media contained Dulbecco’s modified Eagle’s medium/nutrient mixture F-12 media (DMEM/F12; Mediatech Inc.) supplemented with 10% fetal bovine serum (FBS; Sigma-Aldrich), 10% penicillin/streptomycin (Mediatech Inc.), and 100 μg/mL epidermal growth factor (EGF; Sigma-Aldrich). The above digestion step was repeated, and the final filtrate was centrifuged at 3000 rpm for 10 min. The pellet was resuspended in complete AEC media, and AECs were cultured in T75 flasks at approximately 3–5 million cells/flask. The flasks were incubated at 37 °C, 5% CO_2_, and 95% air humidity until they reached 70–80% confluence and were ready to be passaged and treated.

Human AMCs were isolated from the fetal membranes as per the protocol described previously by Sato et al. [77] with slight modifications. Briefly, AECs were removed from the collected fetal membranes by incubation with 0.05% trypsin/EDTA (Corning, Corning, NY, USA) for 1 h in a 37 °C water bath. The remaining membrane was washed 3–4 times in cold HBSS and incubated for 1 h in a rotator in a digestion buffer consisting of Minimum Essential Medium Eagle (Corning), 1 mg/mL collagenase type IV, and 25 µg/mL DNase I. Following complete processing of the membrane, the solution was neutralized with complete AMC culture media. Complete AMC media consisted of DMEM/F12 media supplemented with 5% FBS, 100 U/mL penicillin G, and 10% penicillin/streptomycin. After filtering through a 70 µm cell strainer, the solution was then centrifuged for 10 min at 3000 rpm. The cell pellet was resuspended in complete AMC media, and AMCs were seeded into T75 flasks at a density of 3–5 million cells per flask. The flasks were incubated at 37 °C, 5% CO_2_, and 95% air humidity till they reached 70–80% confluence and were ready to be passaged and treated.

CMCs and CTCs were isolated from fetal membranes as per the protocol described previously by Bailo and Soncini et al. [78] with slight modifications. Briefly, the amnion and chorion parts were peeled off. Decidua was removed from the chorion layer mechanistically. Blood was cleaned off by washing in sterile PBS. The resultant membranes were processed into small pieces in a 2 cm × 2 cm dish under the hood. The fragmented small pieces were subjected to two 8 min incubations in PBS containing 2.4 U/mL dispase at 37 °C, separated by a resting period of 5–10 min in RPMI 1640 medium containing 10% FBS. The stomatal and trophoblastic layers of the chorion were then separated from each other and digested separately in 0.75 mg/mL collagenase solution at 37 °C for 2–3 h. Mobilized cells from the stromal layer, called CMCs, were then collected. The trophoblast side of the chorion was treated further with 0.25% trypsin for 2 min to detach the CTCs, which were also collected. Cells were cultured as discussed above.

### 4.9. Immunofluorescence Staining

AECs were seeded on glass coverslips and stimulated with LPS (100 ng/mL) for 15 min, and cells were fixed with 4% PFA in PBS for 30 min at room temperature and permeabilized with 0.3% Triton in PBS for 15 min. After blocking with 3% BSA in PBS for 2 h, cells were incubated with the primary antibody in a 1:400 dilution of NHERF1 overnight at 4 °C. Cells were stained with antirabbit secondary antibody Alexa Fluor 594 for 1 h. Cells were again washed 3 times with PBST before being mounted with Mowiol 4–88 mounting medium with 4,6-diamidino-2-phenylindole (DAPI). Images were visualized using the Olympus BX43 fluorescent microscope (Olympus, Tokyo, Japan) at 40× magnification, and images were captured using Q Capture Pro software.

### 4.10. Cell Culture Treatments

Cells isolated from the fetal membranes were passage onto T-75 flasks once they reached 70–80% confluence. All experiments were conducted utilizing AECs, AMCs, CMCs, and CTCs at passage 1. Approximately 0.2 × 10^6^ cells were plated for the timepoint assay and cytokine assay. The following treatments were performed in this study: media (control) and lipopolysaccharide (100 ng/mL). The cells were incubated after treatment at 37 °C, 5% CO_2_, and 95% air humidity for different timepoints based on the experiment requirements.

### 4.11. Protein Extraction and Western Blotting

AEC, AMC, CMC, and CTC were lysed with RIPA lysis buffer (50 mM Tris pH 8.0, 150 mM NaCl, 1% Triton X-100, and 1.0 mM EDTA pH 8.0, 0.1% SDS) supplemented with a protease/phosphatase inhibitor cocktail and phenylmethylsulfonyl fluoride, and the protein in the cytoplasmic compartments of the control and LPS-treated primary cells were extracted as described previously by our laboratory. Mouse FM were homogenized as described previously [79]. Protein concentration were determined using Pierce BCA Protein Assay Kit (Thermo Scientific, Waltham, MA, USA). A total of 20 μg of cell lysate/40 μg of tissue lysate was maintained for each sample that was loaded. Western blotting was performed as previously described by our laboratory [79]. Determinations of protein concentrations were done using Pierce BCA Protein Assay Kit (Thermo Scientific, Waltham, MA, USA). The protein concentrations were maintained at the same level for each sample that was loaded (20 μg of cell lysate/40 μg of tissue lysate). Western blotting was performed as previously described by our laboratory [74]. Briefly, samples were run on a gradient (4–15%) of SDS–PAGE Mini-PROTEAN TGX Precast Gels (Bio-Rad, Hercules, CA, USA) and transferred to the membrane using a Bio-Rad Gel Transfer Device (Bio-Rad). Membranes were blocked in 5% nonfat milk in 1× Tris-buffered saline-Tween 20 (TBS-T) for a minimum of 1 h at room temperature before adding the primary antibody and incubating overnight at 4 °C. An appropriate secondary antibody conjugated with horseradish peroxidase was used to incubate the membranes after primary antibody incubation and immunoreactive proteins were visualized using chemiluminescence reagents ECL WB detection system (Amersham Piscataway, NJ, USA). The Restore Western Blot Stripping Buffer (Thermo Fisher) protocol was used before reprobing the blots for β-actin. The antihuman and antimouse antibodies used for WBs were as follows: NHERF1 (1:1000, ab3452, Abcam, USA), NF-kB (1:1000, p-65 NF-kB Ser 348 CST), and β-actin (1:15,000, Sigma- Aldrich, St. Louis, MO, USA). The relative levels of the proteins in the specific bands were normalized with β-actin expression in the same samples, and expressions were densitometrically determined using Bio-Rad Image Lab 6.0 software.

### 4.12. siRNA-Mediated Knockdown of NHERF1 in Fetal Membrane Cells

On-target plus smart pool oligos (Dharmacon, Lafayette, CO, USA) directed against NHERF1 or nontarget scrambled (SCR; control) siRNAs were used to transfect fetal membrane cells (AECs, AMCs, CMCs, and CTCs) using RNAimax lipofectamine per the manufacturer’s instructions. Twenty-four hours after transfection, the cells were used for subsequent assessment of protein expression, stimulation, and cytokine assay.

### 4.13. Statistical Analyses

Statistical analyses for normally distributed data were performed using a Student *t*-test. Statistical values were calculated using GraphPad Prism 8 software (GraphPad Software, Inc., LaJolla, CA, USA). *p* values equal to or less than 0.05 were considered statistically significant. Data in graphs are represented as the mean ± SEM.

## Abbreviations

AEC	amnion epithelial cells
AMC	amnion mesenchymal cells
cAMP	cyclic amino monophosphate
CMC	chorion mesenchymal cells
CTC	chorion trophoblast cells
ERK	extracellular receptor kinase
GPCR	G-protein-coupled receptors
IHC	immunohistochemistry
IL	interleukin
JNK	Janus kinase
LPS	lipopolysaccharide
MMP	Matrix metallopeptidase
NFkB	nuclear factor kappa B
NHERF	sodium hydrogen regulatory factor
PBS	phosphate-buffered saline
pPROM	preterm premature rupture of membranes
PKC	Protein kinase C
PTB	preterm birth
RT-PCR	real-time polymerase chain reaction
siRNA	small interfering RNA
TL	term labor
TNF	tissue necrosis factor
TNIL	term not in labor
